# Gene expression identifies heterogeneity of metastatic behavior among high-grade non-translocation associated soft tissue sarcomas

**DOI:** 10.1186/1479-5876-12-176

**Published:** 2014-06-20

**Authors:** Keith M Skubitz, Amy PN Skubitz, Wayne W Xu, Xianghua Luo, Pauline Lagarde, Jean-Michel Coindre, Frédéric Chibon

**Affiliations:** 1 Department of Medicine, University Hospital, Minneapolis, MN, USA; 2 Masonic Cancer Center, University of Minnesota, Minneapolis, MN, USA; 3 Department of Laboratory Medicine and Pathology, The University of Minnesota Medical School, Minneapolis, MN, USA; 4 The University of Minnesota Supercomputing Institute, Minneapolis, MN, USA; 5 Division of Biostatistics, University of Minnesota School of Public Health, Minneapolis, MN, USA; 6 Institute Bergonie, Bordeaux, France

**Keywords:** Microarray, Sarcoma, Gene expression, Heterogeneity, Subgroups, Metastasis, Prognosis

## Abstract

**Background:**

The biologic heterogeneity of soft tissue sarcomas (STS), even within histological subtypes, complicates treatment. In earlier studies, gene expression patterns that distinguish two subsets of clear cell renal carcinoma (RCC), serous ovarian carcinoma (OVCA), and aggressive fibromatosis (AF) were used to separate 73 STS into two or four groups with different probabilities of developing metastatic disease (PrMet). This study was designed to confirm our earlier observations in a larger independent data set.

**Methods:**

We utilized these gene sets, hierarchical clustering (HC), and Kaplan-Meier analysis, to examine 309 STS, using Affymetrix chip expression profiling.

**Results:**

HC using the combined AF-, RCC-, and OVCA-gene sets identified subsets of the STS samples. Analysis revealed differences in PrMet between the clusters defined by the first branch point of the clustering dendrogram (p = 0.048), and also among the four different clusters defined by the second branch points (p < 0.0001). Analysis also revealed differences in PrMet between the leiomyosarcomas (LMS), dedifferentiated liposarcomas (LipoD), and undifferentiated pleomorphic sarcomas (UPS) (p = 0.0004). HC of both the LipoD and UPS sample sets divided the samples into two groups with different PrMet (p = 0.0128, and 0.0002, respectively). HC of the UPS samples also showed four groups with different PrMet (p = 0.0007). HC found no subgroups of the LMS samples.

**Conclusions:**

These data confirm our earlier studies, and suggest that this approach may allow the identification of more than two subsets of STS, each with distinct clinical behavior, and may be useful to stratify STS in clinical trials and in patient management.

## Background

Soft tissue sarcomas (STS) represent a diverse group of malignancies with different clinical behaviors. Adult STS can be grouped into two broad categories. One category has simple genomic profiles and specific cytogenetic changes, such as a point mutation or translocation (for example SYT-SSX in synovial sarcoma). The second category is comprised of tumors with more complex genomic patterns characterized by multiple gains and losses, including many leiomyosarcomas (LMS), pleomorphic liposarcomas, and undifferentiated pleomorphic sarcomas (UPS) (previously termed malignant fibrous histiocytomas) [1-5]. Although UPS may represent a distinct tumor entity, many UPS have mRNA expression profiles that are similar to other well defined subtypes of STS, including LMS and liposarcoma, although they are not easily recognized as such based on histology (http://www.iarc.fr/en/publications/pdfs-online/pat-gen/bb5/bb5-classifsofttissue.pdf) [6-10].

While some differences in behavior generally correlate with histologic diagnosis and grade, significant heterogeneity of tumor biology exists even within histologic subsets. The heterogeneity of biological behavior complicates clinical care of patients with STS. One clinically important variable is whether a tumor will metastasize or not.

Gene expression patterns may be useful in the subclassification of STS, both for diagnosis and for prediction of clinical behavior [2,7-16]. In some cases, gene expression patterns may correlate better with biological behavior than histology, and some studies have suggested that gene expression patterns may correlate with metastatic potential in some high-grade STS [11,12,14,17]. A recent study identified a set of 67 genes involved in mitosis and chromosome integrity, termed the complexity index in sarcomas (CINSARC), that can predict metastasis outcome in non-translocation dependent STS [11] and also synovial sarcoma [18].

In earlier studies, we described gene expression profiles that identified two general subgroups in a set of clear cell renal cell carcinomas (ccRCC-gene set), a set of ovarian carcinomas (OVCA-gene set), and a set of aggressive fibromatosis samples (AF-gene set) [19-22]. We recently reported the use of a gene set derived from these three studies to separate 73 high grade STS into 2 or 4 groups with different propensity of metastasis [14]. Because the expression data for the STS sample set was limited since it was from a different platform than the Affymetrix system, we pooled the ccRCC-, OVCA-, and AF-gene sets for the earlier study.

In this study we confirmed the results from our earlier studies with an independent data set. We utilized our three gene sets to examine a larger group of 309 non-translocation associated STS using Affymetrix chip based expression profiling, in data sets in which all probes utilized in our earlier studies were represented. These gene sets successfully separated the STS samples into subsets with different probabilities of developing metastases.

## Methods

### Samples

Three hundred nine STS samples were obtained from patients who had surgical resection of STS as previously described [11]. The samples were from the FSG database, part of the Conticabase (http://www.conticabse.org), and were treated in one of 11 centers. In each case, the pathology was reviewed by the pathologist subgroup and classified according to the 2002 WHO classification. For the current study, STS with no recurrent chromosomal translocation were selected. According to French law at the time of the study, experiments were performed in agreement with the Bioethics Law 2004 800 and the Ethics Charter from the National Institute of Cancer; all subjects signed a non-opposition statement for research use of their sample.

### cDNA microarrays

Total RNA was extracted from each frozen tumor sample, and analyzed on Human Genome U133 Plus 2.0 array (Affymetrix) as previously described [11].

### Gene sets

Three different gene sets with limited overlap, that identified two general subgroups of a series of ccRCC samples (ccRCC-gene set), two subgroups of AF (AF-gene set), and distinguished borderline from invasive serous OVCA (OVCA-gene set) have been previously described, including 138, 161, and 173 known genes respectively [19,21,22]. For the current study, these three gene sets were pooled resulting in a set of 533 probes.

### Hierarchical clustering and fold-change analysis

The ccRCC-, AF-, and OVCA- gene sets were used individually or combined, to cluster the 309 primary high-grade STS samples. For clustering, genes were median centered, normalized, and then clustered by complete hierarchical clustering using uncentered correlation with the Eisen clustering software and viewed using the TreeView software (http://rana.lbl.gov). The geometric means of the expression intensities of the relevant gene fragments were computed using Genedata Expressionist (Genedata, Basel, Switzerland), and the ratio was reported as the fold change (up or down). Confidence intervals and p-values on the fold change were calculated using a two-sided Welch modified two-sample t-test. Differences were considered significant when p</=0.05.

### Analysis of time to metastasis

For each data set, we used the Kaplan-Meier (K-M) method to calculate the metastasis-free survival probabilities, and the cumulative probabilities of metastasis (i.e. one minus survival probabilities) at critical time points (2, 3, 5, and 10 years). The p-values were calculated by using the Wilcoxon-Gehan test for comparing different groups. The Tukey-Kramer method was used to adjust for multiple pair-wise comparisons when there were more than two groups. P-values </=0.05 were considered statistically significant. Analyses were performed in SAS® 9.3 (SAS Institute, Cary, NC).

## Results

### Analysis of all STS samples using the combined gene set

**Figure 1:**
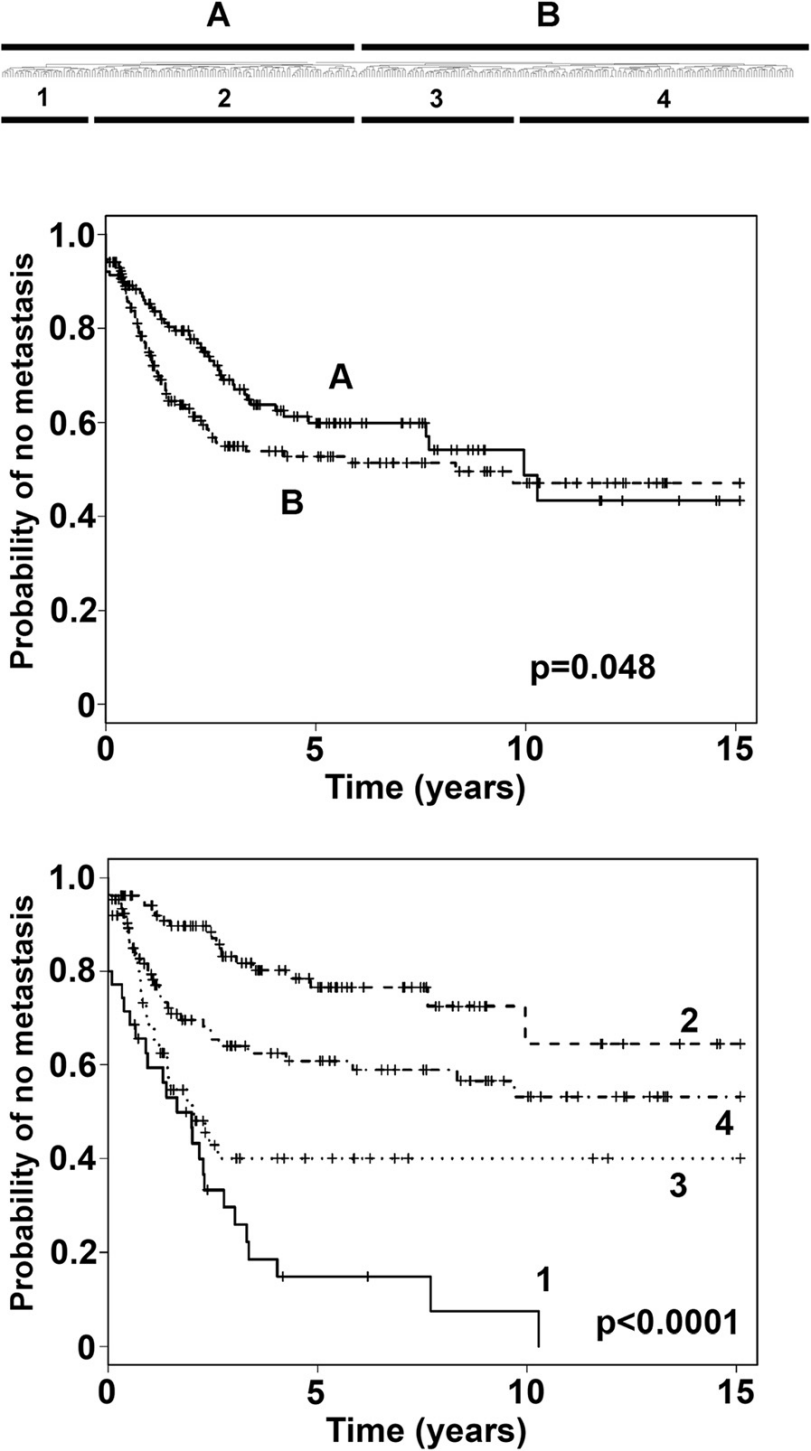
**Top: clustering of gene expression in the 309 high-grade STS samples using the Eisen clustering software Cluster.** The samples were clustered by complete-linkage hierarchical clustering with uncentered correlation using the Eisen clustering software Cluster and the 533 probes present in the pooled gene set as described in the text. Groups **A** and **B** are defined by the first branch point in the clustering. Groups 1–4 are defined by the second branch points. Middle and bottom: Kaplan-Meier analysis of the time to development of metastases of the two groups (groups **A** and **B**) and four groups (groups 1–4), defined by the first and second break points, respectively, of the hierarchical clustering. The time to development of metastasis differed between groups A and B (p = 0.048), and groups 1–4 (p < 0.0001) (see Additional file 1: Table S1).

Hierarchical clustering of the 309 STS samples was first performed with the set of 533 probes of the combined gene set (ccRCC-gene set, OVCA-gene set, and AF-gene set) (Figure 1 top). Kaplan-Meier analysis was performed using the two sample sets defined by the first branch point (Groups A and B). The time to the development of metastasis was significantly different between the two sample sets (p = 0.048), with the probability of not developing a metastasis by 3 years of 0.69 for Group A vs. 0.55 for Group B (Figure 1 middle, Additional file 1: Table S1A).

Kaplan-Meier analysis was also performed using the four sample sets defined by the first and second branch points in the hierarchical clustering analysis (Groups 1–4 in Figure 1 bottom). The time to the development of metastasis differed among the four groups (p < 0.0001), with the probability of remaining free of metastasis by 5 years being 0.15, 0.77, 0.40, and 0.61 for groups 1, 2, 3, and 4, respectively (Figure 1 bottom, Additional file 1: Table S1B). Group comparisons by the Tukey-Kramer method revealed significant differences between groups 1 and 2 (p < 0.0001), 1 and 4 (p = 0.0237), 2 and 3 (p < 0.0001), and 2 and 4 (p = 0.0314).

### Analysis of histologic subtypes

**Figure 2:**
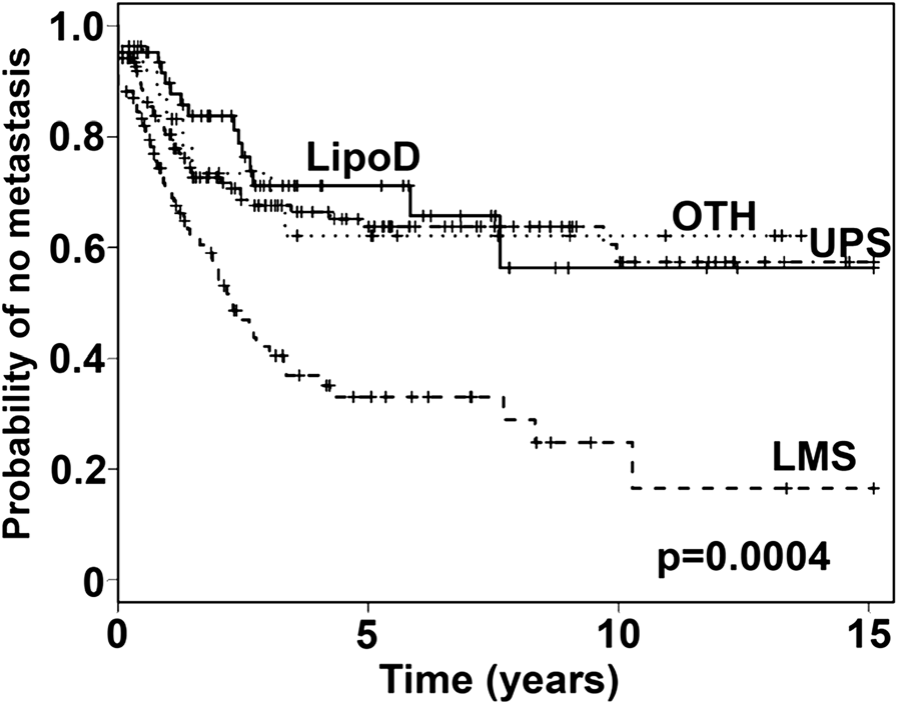
**Kaplan-Meier analysis of the time to development of metastases of the four groups of LMS, LipoD, UPS, and OTH samples.** The time to development of metastasis differed between the groups, p = 0.0004 (see Additional file 1: Table S1).

In examining Figure 1 bottom, we noticed that leiomyosarcoma (LMS) samples were preferentially enriched in group 1, and questioned the impact of differential mixtures of histologic diagnoses to the observed results. We therefore questioned whether there was a difference in probability of metastasis between the LMS, dedifferentiated liposarcoma (LipoD), undifferentiated pleomorphic sarcomas (UPS), and other sarcoma samples (OTH) (which include all other diagnoses as described in reference 11) in our sample set. Analysis revealed that LMS had a higher incidence of metastasis than the other three diagnostic groups (p = 0.0004) (Figure 2, Additional file 1: Table S1C). Group comparisons by the Tukey-Kramer method showed significant differences between LMS and LipoD (p = 0.0004), OTH (p = 0.0016), and UPS (p = 0.0138).

### Analysis of histologic subtypes using the combined gene set

**Figure 3:**
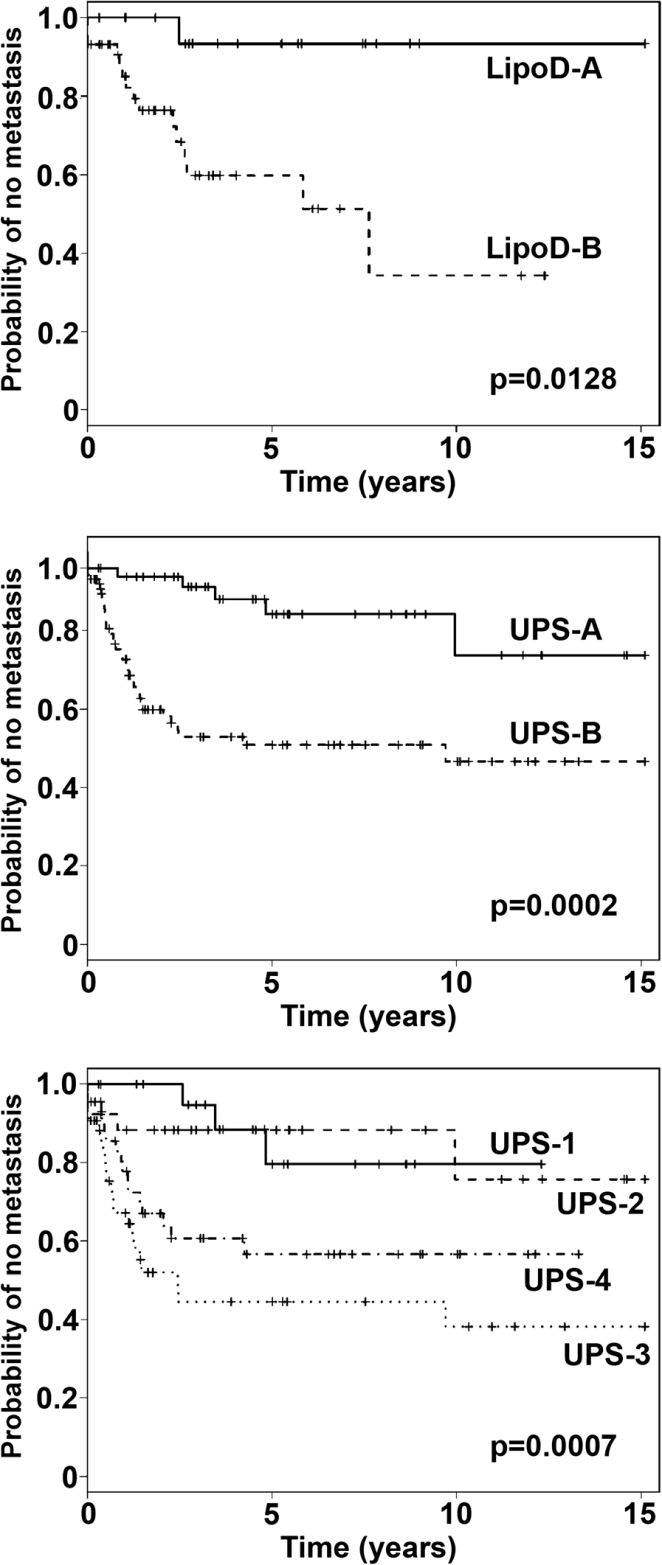
**Kaplan-Meier analysis of the time to development of metastases of the two groups of LipoD samples (groups A and B), defined by the first break point of the hierarchical clustering with the pooled probe set (top panel).** The time to development of metastasis differed between groups **A** and **B**, p = 0.0128. Analysis of the time to development of metastases of the two groups (groups **A** and **B**) (middle panel), and four groups (groups 1–4) of UPS samples (bottom panel) defined by the first and second break points, respectively, of the hierarchical clustering with the pooled probe set. The time to development of metastasis differed between groups **A** and **B**, p = 0.0002, and groups 1–4, p = 0.0007 (see Additional file 1: Table S1).

We next performed hierarchical clustering of the four individual histologic types of STS (LipoD, UPS, LMS, and OTH). Hierarchical clustering of the 62 LipoD samples with the pooled probe set divided the samples into two groups (A and B) with different probabilities of metastasis (Figure 3 top, Additional file 1: Table S1D) (p = 0.0128).

Hierarchical clustering of the 136 UPS samples with the pooled probe set divided the samples into two groups (A and B) with different probabilities of metastasis (Figure 3 middle, Additional file 1: Table S1E) (p = 0.0002). Analysis as four groups (1–4) also revealed statistically significant differences (p = 0.0007) (Figure 3 bottom, Additional file 1: Table S1F), suggesting further heterogeneity in this tumor subset. Group comparisons by the Tukey-Kramer method revealed significant differences between groups 1 and 3 (p = 0.0012), and between groups 2 and 3 (p = 0.0051), while the other group comparisons did not reach statistical significance.

In contrast, when the 84 LMS samples were analyzed in the same manner, analysis of the groups formed by the first 2 or 4 clusters defined by the hierarchical clustering found no statistically significant differences (not shown). Similarly, no difference was observed when a similar analysis was performed using the 27 miscellaneous samples in the OTH subgroup (not shown). Factors possibly contributing to the inability to identify groups with different outcomes in the LMS and OTH sets could include the small number of samples in these two subgroups, and the relative mix of “low” and “high” metastatic samples in these two sample sets.

### Analysis of histologic subtypes using the individual RCC-, OVCA-, and AF-gene sets

**Figure 4:**
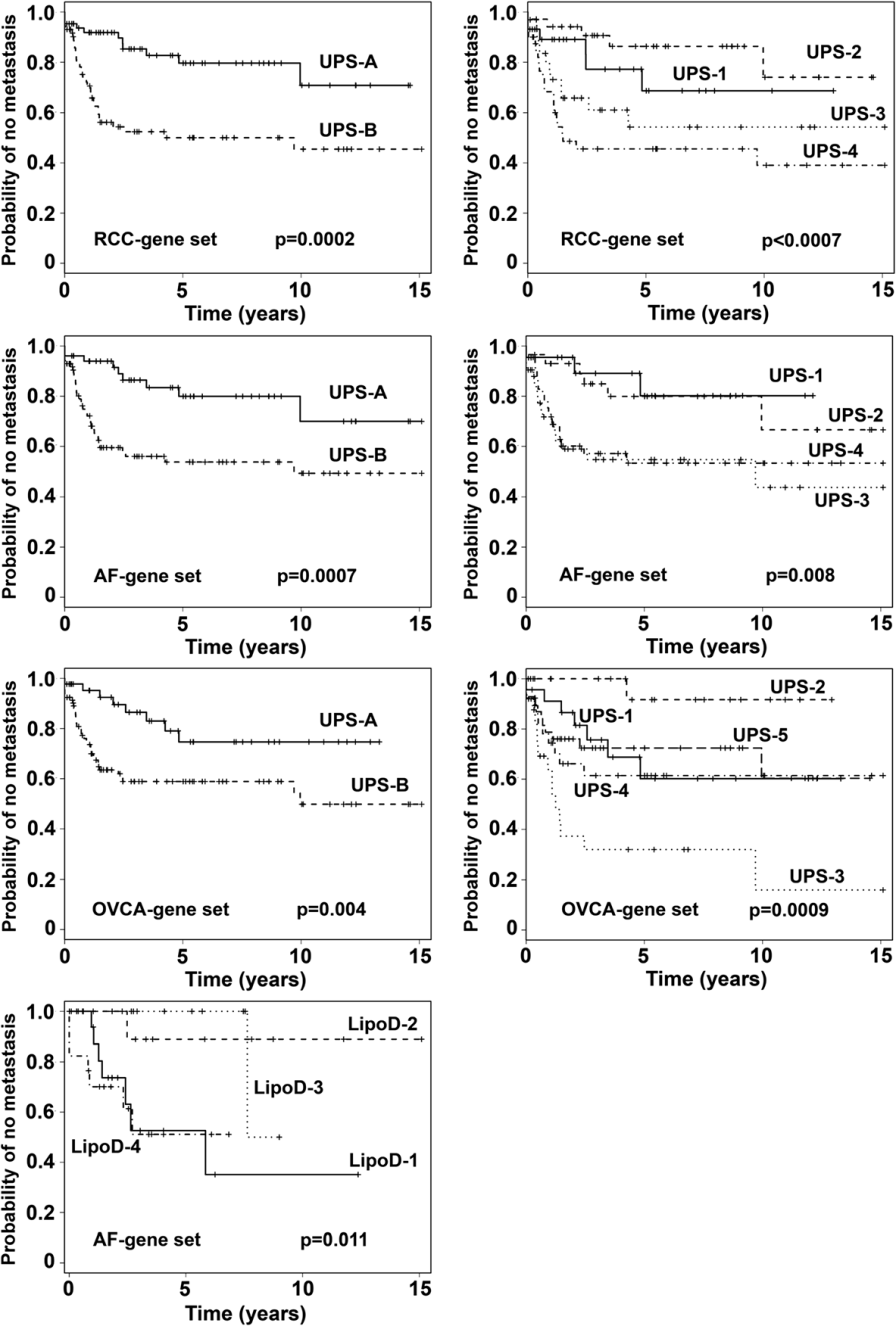
**Top panel: Kaplan-Meier analysis of the time to development of metastases of the two groups (groups A and B) and the four groups (groups 1–4) of UPS samples, defined by the first and second break points of the hierarchical clustering with the RCC-gene set.** The time to development of metastasis differed between groups **A** and **B**, p = 0.0002 and groups 1–4, p < 0.0007 (see Additional file 1: Table S1). Second row: Kaplan-Meier analysis of the time to development of metastases of the two (groups **A** and **B**) and four groups (groups 1–4) of UPS samples defined by the first and second break points, respectively, of the hierarchical clustering with the AF-gene set. The time to development of metastasis differed between groups **A** and **B**, p = 0.0007, and groups 1–4, p = 0.008 (see Additional file 1: Table S1). Third row: Kaplan-Meier analysis of the time to development of metastases of the two groups (groups **A** and **B**), and five groups (groups 1–5) of the UPS samples, defined by the first break point, and visual separation of the break points, respectively, of the hierarchical clustering with the OVCA-gene set. The time to development of metastasis differed between groups **A** and **B**, p = 0.004, and between groups 1–5, p = 0.0009 (see Additional file 1: Table S1). Bottom row: Kaplan-Meier analysis of the time to development of metastases of the four groups of LipoD samples defined by the second break points of the hierarchical clustering with the AF-gene set, p = 0.011 (see Additional file 1: Table S1).

We also analyzed the STS samples with the individual AF-, OVCA-, and RCC-probe sets. Analysis of the UPS subset with the RCC-gene set identified differences in time to metastasis when the UPS samples were analyzed as two groups (p = 0.0002) or four groups (p < 0.0007)(Figure 4, top row, Additional file 1: Table S1 G,H). Group comparisons by the Tukey-Kramer method revealed significant differences between groups 1 and 4 (p = 0.0159) and 2 and 4 (p = 0.0005) with a trend between 2 and 3 (p = 0.0971). Analysis of the UPS samples with the AF-gene set revealed differences in time to metastasis when analyzed as two groups (p = 0.0007) or four groups (p = 0.008) (Figure 4, second row, Additional file 1: Table S1 I,J). Group comparisons by the Tukey-Kramer method revealed significant differences between groups 1 and 3 (p = 0.0500), and 2 and 3 (p = 0.0422), with a trend between groups 2 and 4 (p = 0.1196) and 1 and 4 (p = 0.1451). Analysis of the UPS samples with the OVCA-gene set found differences in time to metastasis when the samples were analyzed as two (p = 0.004), or five subgroups (Figure 4, third row, Additional file 1: Table S1, K,L) (p = 0.0009). Group comparisons by the Tukey-Kramer method revealed significant differences between groups 1 and 3 (p = 0.0303) and 2 and 3 (p = 0.0004).

When the AF-gene set was used to cluster the LipoD samples and analyzed as two groups the difference in time to metastasis did not reach statistical significance (not shown). Although when analyzed as four subgroups, statistically significant differences in time to metastasis were observed (Figure 4 bottom, Additional file 1: Table S1 M) (p = 0.011). Group comparisons by the Tukey-Kramer method revealed significant differences between groups 2 and 4 (p = 0.0384) and 3 and 4 (p = 0.0220). When either the RCC-gene set or the OVCA-gene set was used to cluster the LipoD samples, no significant difference in time to metastasis was noted when analyzed as 2, 4, or 5 groups, although a trend was suggested (not shown). None of the individual AF-, RCC-, or OVCA- gene sets separated the LMS or OTH samples into groups of different metastatic propensity.

### Effect of tumor grade on probability of developing metastases

Tumor grade is correlated with survival in STS, and we therefore questioned whether this might contribute to our observations. We performed Kaplan-Meier analyses of the LipoD and UPS subgroups defined by clustering with the total gene set as in Figure 3 top and middle, but excluded the 7 LipoD and 7 UPS samples that were histologically grade 1 tumors. In the case of the LipoD samples, the samples present in the first two groups defined by clustering still differed in the probability of developing metastases (p = 0.0545) (not shown). Similarly, when the UPS samples were analyzed without grade 1 tumors, a difference in probability of metastasis was observed when analyzed as two groups (p = 0.0004) or four groups (p = 0.001) (not shown). Group comparisons using the Tukey-Kramer method found significant differences between groups 1 and 3 (p = 0.002) and 2 and 3 (p = 0.006). Therefore, the subgroups identified by clustering in Figure 3 were not accounted for by differences in content of grade 1 tumors.

### Effect of stage on probability of developing metastasis

Since clinical grade is correlated with survival in STS, we questioned whether this might contribute to our observations. When the LipoD samples were analyzed by AJC (American Joint Commission) stage, no significant difference in the probability of developing metastases was seen between stage III (n = 17) vs IIA (n = 2), III vs IIB (n = 26), or IIA vs IIB, though the difference between stage III and IIA trended toward significance at p = 0.15 by Tukey-Kramer, and the rank order was as expected. When the UPS samples were analyzed by stage (IIA, n = 23; IIB, n = 30; III, n = 65), the three stages also did not differ in risk of metastases, though the difference between stage IIA and III and that between stage IIB and III also trended to significance, both at p = 0.07, and the rank order was again as expected. The lack of good correlation between stage and metastases seen here may reflect the limited number of cases examined in the different stages, and the fact that most cases were either IIA, IIB, or III.

### Correlations between AJC stage and clustering

For the UPS samples, AJC stage was significantly correlated with both the two groups and four groups defined by clustering (Figure 3, middle and bottom) (p = 0.03 and 0.02, respectively). For the LipoD samples, AJC stage was not significantly correlated with the two groups formed by clustering (Figure 3, top).

### Differential gene expression between subgroups

We next examined the most differentially expressed genes of the total detected in the Affymetrix U133 Plus chip set, in the first two subsets (groups A and B) of LipoD and UPS (Figure 3, top and middle). Fold change analysis was performed as described in the Methods, and the genes most over- or under-expressed between the groups are shown for LipoD in Additional file 2: Table S2 and for UPS in Additional file 3: Table S3.

When the global gene expression of the LipoD-A subset was compared with the LipoD-B subset (Figure 3 top), many differences were found (Additional file 2: Table S2). For example, in the LipoD-A subset (the better prognosis group) several genes that would be expected to be expressed in adipocytes, including: ADIPOQ, PLIN1, FABP4, LPL, PPARG, and THRSP were over-expressed compared with the LipoD-B subset, as well as ALDH1, ADH1B, and NTRK2 (Additional file 2: Table S2A). This may correlate with a less “de-differentiated” state. In contrast, genes over-expressed in the LipoD-B subset (the worse prognosis group) compared to the LipoD-A subset included: RUNX2, CDC20, TOP2A, ANLN, multiple members of the kinesin family, CDK1, AURKA, CCNB1, CDKN3, LOX, CKS2, CCNA2, PDPN, AURKB, CCNE2, and the extracellular matrix genes FN1 and CTHRC1 (Additional file 2: Table S2B).

The most over-expressed gene in the UPS-A subset (the good prognosis group) was SCARA5, scavenger receptor class A, member 5, a gene that may act as a tumor suppressor in some models [23]. Other genes over-expressed in UPS-A compared to UPS-B included: TNXB, ALDH1A3, ADA1B, MFAP5, AKAP12, DEFB1, TGFBR3, GAS7, IL17D, IL33, and CD34, as well as the growth factors NEGR1, PDGFD, FGF18, NTRK2, and IGFBP6, a regulator of IGF1 (Additional file 3: Table S3A). In contrast, genes over-expressed in the UPS-B subset (the bad prognosis group) compared with the UPS-A subset included: STEAP1, CA12, AIM2, TNC, HS3ST3A1, RUNX2, POSTN, ADAM12, PLAUR, NRP2, RGS1, extracellular matrix proteins COL11A1, COL10A1, and FN, matrix metalloproteinases MMP9, MME, MMP13, and MMP1, FAP (a marker of fibroblast activation), and growth regulator proteins (Additional file 3: Table S3B).

Several genes were upregulated in both UPS-B and LipoD-B (the poor prognosis groups) relative to UPS-A and LipoD-A, respectively (Additional file 4: Table S4). Only one of these genes, RRM2, is present in the CINSARC gene set [11]. Five of these genes, RRM2, LEF1, KDELR3, FN1, and CTHRC1, were present in the RCC-gene set; two genes, MICAL2 and KDELR3, were present in the AF-gene set; and one gene, RRM2, was present in the OVCA-gene set.

## Discussion

In the current study, 309 cases of four histologic subtypes (LipoD, UPS, LMS, OTH) of non-translocation associated soft tissue sarcoma (STS) were separated into subsets with different probability of metastasis using gene sets derived from our earlier studies with ccRCC, AF, and OVCA [15,19-22]. Each of the three gene sets separated the 136 UPS samples into two groups of differing metastatic propensity, while the AF-gene set also identified four subsets of the 62 LipoD samples. These data support the concept that these gene sets may predict biological behavior in some STS. The data also confirmed differences in the metastatic propensity between LMS and the other STS examined.

Both the current and CINSARC gene sets identified subsets of the UPS samples, when analyzed as a separate group, that differed in time to metastasis. Interestingly, the current study also detected subsets of LipoD, when analyzed as a separate group, that differed in probability of metastasis, but did not detect such subsets in the LMS or OTH subgroups. These differences were apparent even after excluding grade 1 tumors from the analysis. In contrast, the CINSARC gene set identified subsets of LMS, when analyzed as a separate group, that differed in probability of metastasis, but did not detect such subsets in the LipoD subgroup [11]. Copy number losses, which may also reflect genomic instability, have recently been shown to identify subgroups of LipoD with a poor prognosis [24]. These results are consistent with the hypothesis that gene expression patterns that correlate with metastatic propensity may depend on the background gene expression of the tumor, which may be determined by other gene sets and partly reflected by histology. For example, a set of genes that predicts metastasis in LMS might not be predictive in UPS, or a gene might be predictive in lung cancer but not colon cancer.

Differences were observed in the global gene expression patterns of the subsets that were identified in this study. For example, the subset of the LipoD samples with the lower probability of metastasis (LipoD-A) tended to over-express a number of genes expressed in adipocytes such as ADIPOQ, PLIN1, FABP4, LPL, PPARG, and THRSP. These samples might be viewed as less de-differentiated. Adiponectin, encoded by the ADIPOQ gene, is a hormone produced in adipose tissue that regulates several metabolic processes including fatty acid catabolism. It is strongly expressed in preadipocytes differentiating to adipocytes [25,26]. Perilipin, also known as lipid droplet-associated protein, is encoded by the PLIN1 gene and coats lipid droplets in adipocytes, acting as a coating that separates lipids from lipases. FABP4, also known as adipocyte protein 2, is a carrier protein for fatty acids and is expressed in adipocytes and macrophages. Lipoprotein lipase (LPL) is expressed in a number of tissues; the form in adipocytes is activated by insulin. PPARG, or peroxisome proliferator-activated receptor gamma, is a nuclear receptor that regulates fatty acid storage and glucose metabolism. PPARG activates genes that stimulate lipid uptake and adipogenesis by fat cells. THRSP, or thyroid hormone responsive SPOT14, plays an important role in regulating lipid metabolism [27]. Interestingly, SPOT14 has been reported to be a marker of aggressive breast cancer [28].

Other genes that were over-expressed in the LipoD-A subset of samples included ALDH1, ADH1B, and NTRK2. Alcohol dehydrogenase 1B (ADH1B) metabolizes a variety of substrates. Aldehyde dehydrogenase 1 (ALDH1) oxidizes aldehydes to carboxylic acids and also metabolizes a variety of substrates. ALDH1 is a member of a superfamily of genes and has been reported to be a marker of normal and malignant mammary stem cells, and a predictor of poor clinical outcome in breast cancer [29]. NTRK2 functions as a receptor for several neurotrophins, including BDNF, NT-3, and NT-4; it can mediate several effects, including differentiation and survival.

In contrast, the LipoD-B subset (with the higher risk of developing metastatic disease) over-expressed a number of genes involved in regulating cell growth and cell division. Among the genes over-expressed in the LipoD-B subset compared to the LipoD-A subset, RUNX2 is a transcription factor that has a Runt DNA-binding domain and is active in osteoblast differentiation. CDC20 (cell-division cycle protein 20), cyclin B1 (CCNB1), cyclin A2 (CCNA2), cyclin E2 (CCNE2), cyclin-dependent kinase-1 (CDK1, also known as CDC2), cyclin-dependent kinases regulatory subunit 2 (CKS2), and cyclin-dependent kinase inhibitor 3 (CDKN3), aurora A kinase (AURKA), and aurora B kinase (AURKB) are involved in regulating cell division. TOP2A (DNA topoisomerase 2-alpha) is important in DNA replication and is felt to be a target for doxorubicin and etoposide. In some models, higher levels of TOP2A correlated with more resistance to doxorubicin [30]. Podoplanin (PDPN) is a mucin-like protein expressed in a variety of cells. Notably, PDPN is a specific marker for lymphatic endothelial cells, and has been shown to be over-expressed in a variety of tumors. ANLN (actin binding protein anillin) binds actin, but is also localized to the nucleus in some cancer cells, and has been reported to be over-expressed in hormone resistant prostate cancer and squamous cell head and neck cancer. Lysyl oxidase (LOX) is a copper containing enzyme that catalyzes the formation of aldehydes from lysine, especially in collagen and elastin [31]. LOX is regulated by hypoxia-inducible factors (HIFs) and has been reported to be upregulated in a number of cancers. In a mouse model, LOX inhibitors decreased metastasis [32] and such inhibitors could eventually be clinically useful [33]. Many of these regulatory proteins could serve as drug targets.

Extracellular matrix genes were also differentially expressed in the two LipoD subsets, with FN1 and CTHRC1 over-expressed in LipoD-B. Differential expression of extracellular matrix proteins in different subsets of malignancies appears to be a common theme. The recognition of a desmoplastic response to cancer has long been recognized [34-36], and tumor-associated fibroblasts may play a role in cancer growth and development [37-42]. The expression of several extracellular matrix and collagen genes has been related to invasion and survival in other tumors [43-45], and studies in breast cancer described two types of stromal responses: a fibromatosis-like stromal gene signature and a CSF-1 macrophage stromal gene signature [46].

Genes over-expressed in UPS-A (the good prognosis group) compared to UPS-B included: SCARA5, TNXB, ALDH1A3, ADH1B, MFAP5, AKAP12, DEFB1, TGFBR3, GAS7, IL17D, IL33, and CD34, as well as the growth factors NEGR1, PDGFD, FGF18, NTRK2, and IGFBP6, a regulator of IGF1. SCARA5 was expressed 34-fold higher in the UPS-A subgroup, and may function as a tumor suppressor gene. Over-expression of SCARA5 suppressed some malignant behaviors in hepatoma cells, and SCARA5 knockdown was associated with activation of MMP9 [23]. Tenascin-XB (TNXB) is an extracellular matrix protein that may regulate collagen deposition by fibroblasts. Immune related proteins over expressed in UPS-A included: DEFB1 (defensin beta 1), IL17D, IL33, and A-kinase anchor protein 12 (AKAP12).

In contrast, genes over-expressed in UPS-B (the bad prognosis group) compared to UPS-A (the good prognosis group) included: STEAP1, CA12, AIM2, TNC, HS3ST3A1, RUNX2, POSTN, ADAM12, PLAUR, NRP2, RGS1, extracellular matrix proteins (COL11A1, COL10A1, FN), matrix metalloproteinases (MMP9, MME, MMP13, MMP1), FAP (a marker of fibroblast activation), and growth regulator proteins. Six-transmembrane epithelial antigen of the prostate (STEAP1) is an antigen detected in prostate cells, but up-regulated in multiple cancer cell lines, and is a metalloreductase. Recent studies on the expression patterns of STEAP1 and STEAP2 have suggested that they may be markers of mesenchymal stem cells [47]. High STEAP1 expression in Ewing sarcoma has been reported to be associated with an improved outcome [48]. In contrast, STEAP1 expression has also been reported to promote invasiveness in Ewing tumors [49]. PLAUR has been associated with cell migration, cell adhesion, and cell cycle regulation.

Of the genes upregulated in both of the poor-prognosis groups (UPS-B and LipoD-B) compared with UPS-A and LipoD-A, respectively, only RRM2 is present in the CINSARC gene set; five genes, RRM2, LEF1, KDELR3, FN1, and CTHRC1, were present in the RCC-gene set; two genes, MICAL2 and KDELR3, were present in the AF-gene set; and one gene, RRM2, was present in the OVCA-gene set. The RRM2 gene encodes ribonucleoside-diphosphate reductase subunit M2, one of two subunits for ribonucleotide reductase. Ribonucleotide reductase catalyzes the formation of deoxyribonucleotides from ribonucleotides, a rate-limiting step in DNA synthesis, and is regulated in a cell-cycle dependent manner. Over-expression of RRM2 has been reported to enhance the metastatic potential of some tumors [50]. LEF1 is a transcription factor involved in the Wnt-signaling pathway, and has been associated with other malignancies. KDELR3 is a receptor involved in protein sorting in the endoplasmic reticulum. MICAL2 is a monooxygenase that promotes depolymerization of F-actin, and has also been associated with progression of prostate cancer.

Although in the current study we focused on the identification of at most four subsets of each STS set with different biological behavior (manifest as metastatic propensity), in some cases we observed statistically significant differences in time to metastasis when the samples were analyzed as more than four subsets. The ability to detect multiple subgroups is strongly influenced by sample number and the distribution of samples among the various groups. Perhaps further heterogeneity that is clinically useful may be identified in larger sample sets. The ability to better predict the long-term outcome following surgery will greatly improve the treatment of patients with sarcomas, and gene expression profiles may provide a clinically meaningful approach to this problem, restricting the use of adjuvant modalities of therapy and reducing heterogeneity among groups in clinical trials of new drugs.

## Conclusions

The biologic heterogeneity of soft tissue sarcomas (STS), even within histological subtypes, complicates treatment. This study used hierarchical clustering with a gene set derived from studies of renal carcinoma, ovarian carcinoma, and aggressive fibromatosis, to examine 309 STS using Affymetrix chip expression profiling. The analysis separated STS into two groups, and also four groups, with different probabilities of developing metastatic disease (PrMet). Analysis also revealed differences in PrMet between leiomyosarcomas (LMS), dedifferentiated-liposarcomas (LipoD), and undifferentiated pleomorphic sarcomas (UPS). HC of both the LipoD and UPS samples divided the samples into two groups with different PrMet. HC of the UPS samples also showed four groups with different PrMet. These data confirm our earlier studies in with a different sample set, and suggest that this approach may allow the identification of more than two subsets of STS, each with distinct clinical behavior, and may be useful to stratify STS in clinical trials and in patient management.

## Abbreviations

STS: Soft tissue sarcomas; RCC: Renal cell carcinoma; OVCA: Ovarian cancer; AF: Aggressive fibromatosis; HC: Hierarchical clustering; PrMet: Probability of developing metastatic disease; LMS: Leiomyosarcoma; LipoD: Dedifferentiated liposarcoma; UPS: Undifferentiated pleomorphic sarcoma.

## Competing interests

The authors declare that they have no competing interests.

## Authors’ contributions

KMS participated in study design, data analysis, and helped draft the manuscript. APNS participated in study design, data analysis, and helped draft the manuscript. WX participated in data analysis, and helped draft the manuscript. XL participated in data analysis, and helped draft the manuscript. PL participated in data acquisition and analysis, and helped draft the manuscript. JMC participated in data acquisition and analysis, and helped draft the manuscript. FC participated in data acquisition and analysis, and helped draft the manuscript. All authors read and approved the final manuscript.

## Supplementary Material

Additional file 1: Table S1 Probability (95% CI) of no metastasis as a function of time. Click here for file

Additional file 2 Genes over-expressed in LipoD-A vs LipoD-B. Click here for file

Additional file 3 Genes over-expressed in UPS-A vs UPS-B. Click here for file

Additional file 4 Genes over-expressed in both LipoD-B vs LipoD-A and UPS-B vs UPS-A. Click here for file
