# Supplementary material for: Gene expression identifies heterogeneity of metastatic behavior among high-grade non-translocation associated soft tissue sarcomas
Source: J Transl Med. 2014 Jun 20;12:176. doi: 10.1186/1479-5876-12-176 (PMC4082412; doi:10.1186/1479-5876-12-176)
Supplement: Additional file 1: Table S1 — Probability (95% CI) of no metastasis as a function of time. [file 1479-5876-12-176-S1.pdf]

Table 1. Probability (95% CI) of no metastasis as a function of time.

A. The two sample subsets defined in Figure 1, middle (p=0.048).

Time (years)	2	3	5	10
Group A n=139	0.79 (0.70-0.85)	0.69 (0.60-0.77)	0.60 (0.50-0.69)	0.49 (0.34-0.62)
Group B n=170	0.63 (0.55-0.70)	0.55 (0.46-0.63)	0.53 (0.44-0.61)	0.47 (0.37-0.57)

B. The four sample subsets defined in Figure 1, bottom (p<0.0001).

Time (years)	2	3	5	10
Group 1 n=35	0.50 (0.32-0.65)	0.30 (0.15-0.46)	0.15 (0.05-0.30)	0.07 (0.01-0.25)
Group 2 n=104	0.90 (0.82-0.94)	0.83 (0.73-0.90)	0.77 (0.65-0.85)	0.64 (0.43-0.79)
Group 3 n=62	0.52 (0.39-0.65)	0.40 (0.26-0.54)	0.40 (0.26-0.54)	0.40 (0.26-0.54)
Group 4 n=108	0.70 (0.59-0.78)	0.64 (0.53-0.73)	0.61 (0.49-0.70)	0.53 (0.40-0.65)

C. The four sample subsets defined by histology shown in Figure 2 (p=0.0004).

Time (years)	2	3	5	10
LMS n=84	0.56 (0.44-0.66)	0.42 (0.30-0.53)	0.33 (0.22-0.45)	0.25 (0.13-0.39)
LipoD n=62	0.84 (0.71-0.91)	0.71 (0.56-0.82)	0.71 (0.56-0.82)	0.56 (0.32-0.75)
Other n=27	0.73 (0.50-0.87)	0.73 (0.50-0.87)	0.62 (0.38-0.79)	0.62 (0.38-0.79)
UPS n=136	0.73 (0.64-0.80)	0.68 (0.58-0.75)	0.64 (0.54-0.72)	0.57 (0.45-0.68)

D. The two LipoD sample subsets defined in Figure 3, top (p=0.0128).

Time (years)	2	3	5	10
Group A n=18	1.00 (NA)	0.93 (0.61-0.99)	0.93 (0.61-0.99)	0.93 (0.61-0.99)
Group B n=44	0.76 (0.59-0.87)	0.60 (0.40-0.75)	0.60 (0.40-0.75)	0.34 (0.08-0.63)

E. The two UPS sample subsets defined in Figure 3, middle (p=0.0002).

Time (years)	2	3	5	10
Group A n=49	0.94 (0.82-0.98)	0.91 (0.78-0.97)	0.84 (0.67-0.93)	0.74 (0.44-0.89)
Group B n=87	0.60 (0.48-0.70)	0.53 (0.41-0.64)	0.51 (0.38-0.62)	0.47 (0.33-0.59)

F. The four UPS sample subsets defined in Figure 3, bottom (p=0.0007).

Time (years)	2	3	5	10
Group 1 n=23	1.00 (NA)	0.95 (0.68-0.99)	0.80 (0.48-0.93)	0.80 (0.48-0.93)
Group 2 n=26	0.88 (0.68-0.96)	0.88 (0.68-0.96)	0.88 (0.68-0.96)	0.76 (0.40-0.92)
Group 3 n=43	0.52 (0.35-0.67)	0.44 (0.27-0.60)	0.44 (0.27-0.60)	0.38 (0.20-0.56)
Group 4 n=44	0.67 (0.50-0.79)	0.61 (0.43-0.74)	0.57 (0.39-0.71)	0.57 (0.39-0.71)

G. The two UPS sample subsets defined in Figure 4, top row with the RCC-gene set (p=0.0002).

Time (years)	2	3	5	10
Group A	0.92 (0.81-0.97)	0.85 (0.72-0.92)	0.80 (0.65-0.89)	0.71 (0.47-0.86)
Group B	0.56 (0.43-0.67)	0.52 (0.39-0.64)	0.50 (0.37-0.62)	0.45 (0.31-0.59)

H. The four UPS sample subsets defined in Figure 4, top row with the RCC-gene set (p<0.0007).

Time (years)	2	3	5	10
Group 1 n=29	0.90 (0.70-0.96)	0.77 (0.52-0.90)	0.69 (0.40-0.86)	0.69 (0.40-0.86)
Group 2 n=35	0.94 (0.78-0.98)	0.91 (0.74-0.97)	0.86 (0.67-0.95)	0.74 (0.40-0.91)
Group 3 n=32	0.66 (0.46-0.80)	0.61 (0.40-0.76)	0.54 (0.32-0.72)	0.54 (0.32-0.72)
Group 4 n=40	0.49 (0.32-0.64)	0.46 (0.29-0.61)	0.46 (0.29-0.61)	0.39 (0.21-0.56)

I. The two UPS sample subsets defined in Figure 4, second row with the AF-gene set (p=0.0007).

Time (years)	2	3	5	10
Group A n=51	0.94 (0.82-0.98)	0.86 (0.72-0.94)	0.80 (0.63-0.90)	0.70 (0.43-0.86)
Group B n=85	0.59 (0.47-0.70)	0.56 (0.44-0.67)	0.54 (0.41-0.65)	0.49 (0.35-0.62)

J. The four UPS sample subsets defined in Figure 4, second row with the AF-gene set (p=0.008).

Time (years)	2	3	5	10
Group 1 n=22	0.95 (0.72-0.99)	0.89 (0.62-0.97)	0.80 (0.48-0.94)	0.80 (0.48-0.94)
Group 2 n=29	0.93 (0.75-0.98)	0.85 (0.64-0.94)	0.80 (0.58-0.91)	0.67 (0.33-0.86)
Group 3 n=42	0.59 (0.41-0.73)	0.55 (0.36-0.70)	0.55 (0.36-0.70)	0.44 (0.20-0.65)
Group 4 n=43	0.60 (0.43-0.73)	0.57 (0.40-0.71)	0.53 (0.36-0.68)	0.53 (0.36-0.68)

K. The two UPS sample subsets defined in Figure 4, third row with the OVCA-gene set (p=0.004).

Time (years)	2	3	5	10
Group A n=44	0.92 (0.78-0.98)	0.86 (0.70-0.94)	0.75 (0.55-0.87)	0.75 (0.55-0.87)
Group B n=92	0.63 (0.52-0.73)	0.59 (0.47-0.69)	0.59 (0.47-0.69)	0.50 (0.34-0.63)

L. The five UPS sample subsets defined in Figure 4, third row with the OVCA- gene set (p=0.0009).

Time (years)	2	3	5	10
Group 1 n=23	0.87 (0.64-0.95)	0.76 (0.51-0.89)	0.60 (0.32-0.80)	0.60 (0.32-0.80)
Group 2 n=21	1.00 (NA)	1.00 (NA)	0.92 (0.54-0.99)	0.92 (0.54-0.99)
Group 3 n=25	0.37 (0.17-0.58)	0.32 (0.13-0.52)	0.32 (0.13-0.52)	0.16 (0.01-0.45)
Group 4 n=28	0.66 (0.45-0.81)	0.61 (0.40-0.77)	0.61 (0.40-0.77)	0.61 (0.40-0.77)
Group 5 n=39	0.76 (0.59-0.87)	0.72 (0.54-0.84)	0.72 (0.54-0.84)	0.60 (0.32-0.80)

M. The four LipoD sample subsets defined in Figure 4, bottom row with the AF-gene set (p=0.011).

Time (years)	2	3	5	10
Group 1 n=22	0.74 (0.44-0.89)	0.53 (0.22-0.76)	0.53 (0.22-0.76)	0.35 (0.07-0.66)
Group 2 n=12	1.00 (NA)	0.89 (0.43-0.98)	0.89 (0.43-0.98)	0.89 (0.43-0.98)
Group 3 n=11	1.00 (NA)	1.00 (NA)	1.00 (NA)	Insufficient data
Group 4 n=17	0.70 (0.42-0.86)	0.51 (0.22-0.74)	0.51 (0.22-0.74)	Insufficient data
