# Supplementary material for: Gene expression identifies heterogeneity of metastatic behavior among high-grade non-translocation associated soft tissue sarcomas
Source: J Transl Med. 2014 Jun 20;12:176. doi: 10.1186/1479-5876-12-176 (PMC4082412; doi:10.1186/1479-5876-12-176)
Supplement: Additional file 2 — Genes over-expressed in LipoD-A vs LipoD-B. [file 1479-5876-12-176-S2.zip › Table 2A.pdf]

Table 2A	Genes over-expressed in LipoD-A vs LipoD-B	
Probe id	Gene Symbol	Fold Change (Up in LipoD-A vs LipoD-B)
207175_at	ADIPOQ	167.9
205913_at	PLIN1	150.8
229476_s_at	THRSP	120.9
235978_at	FABP4	83.0
229477_at	THRSP	75.0
203548_s_at	LPL	72.5
209612_s_at	ADH1B	57.6
219398_at	CIDEC	56.1
208383_s_at	PCK1	55.7
209613_s_at	ADH1B	55.2
228409_at	PLIN4	48.0
203549_s_at	LPL	47.3
219140_s_at	RBP4	44.4
203980_at	FABP4	44.4
213706_at	GPD1	42.9
205478_at	PPP1R1A	41.4
203680_at	PRKAR2B	39.8
224327_s_at	DGAT2	39.0
1552509_a_at	CD300LG	35.1
211708_s_at	SCD	33.4
204997_at	GPD1	30.6
211162_x_at	SCD	27.9
212741_at	MAOA	27.2
221796_at	NTRK2	27.0
221795_at	NTRK2	26.8
1554044_a_at	MRAP	24.2
202768_at	FOSB	24.0
213524_s_at	G0S2	23.9
49452_at	ACACB	23.9
203571_s_at	C10orf116	23.6
1555740_a_at	MRAP	23.4
209555_s_at	CD36	23.3
218087_s_at	SORBS1	23.2
206243_at	TIMP4	22.9
223727_at	KCNIP2	22.1
1553583_a_at	THRSP	21.7
231736_x_at	MGST1	21.2
244276_at	KLB	20.6
219689_at	SEMA3G	20.5
226064_s_at	DGAT2	20.4
241929_at	---	20.2
224918_x_at	MGST1	19.9
223828_s_at	LGALS12	19.4
209616_s_at	CES1	19.2
209686_at	S100B	19.1
228766_at	CD36	18.5
221295_at	CIDEA	18.2
205498_at	GHR	18.1
43427_at	ACACB	18.1
209763_at	CHRDL1	17.9

219064_at	ITIH5	17.7
242736_at	---	17.6
214033_at	ABCC6	17.3
1565162_s_at	MGST1	17.2
215039_at	HS2ST1 /// LOC	17.1
210964_s_at	GYG2	16.1
222513_s_at	SORBS1	16.0
205220_at	GPR109B	15.8
220161_s_at	EPB41L4B	15.6
239262_at	---	15.4
206488_s_at	CD36	15.2
215559_at	ABCC6 /// LOC	15.1
229004_at	ADAMTS15	15.0
200831_s_at	SCD	14.9
205382_s_at	CFD	14.7
1552519_at	ACVR1C	14.7
204041_at	MAOB	14.1
221928_at	ACACB	13.9
204894_s_at	AOC3	13.6
207092_at	LEP	13.6
225207_at	PDK4	12.9
208186_s_at	LIPE	12.8
228568_at	GCOM1	12.7
243879_at	---	12.7
208510_s_at	PPARG	12.3
1555854_at	AKR1C1 /// AKR	12.2
214433_s_at	SELENBP1	12.1
231050_at	HRASLS5	11.9
235708_at	KLB	11.9
206955_at	AQP7	11.8
205428_s_at	CALB2	11.6
243584_at	---	11.4
222317_at	PDE3B	11.1
228268_at	FMO2	10.9
204039_at	CEBPA	10.8
215695_s_at	GYG2	10.8
209614_at	ADH1B	10.8
204388_s_at	MAOA	10.7
228434_at	BTNL9	10.4
1558421_a_at	C14orf180	10.3
210963_s_at	GYG2	10.1
235670_at	STX11	10.0
206209_s_at	CA4	9.9
230463_at	---	9.9
203296_s_at	ATP1A2	9.8
219295_s_at	PCOLCE2	9.7
205960_at	PDK4	9.6
205440_s_at	NPY1R	9.5
1562275_at	---	9.2
238066_at	RBP7	9.0
202921_s_at	ANK2	8.9
214091_s_at	GPX3	8.8
1552616_a_at	ACACB	8.8

214461_at	LBP	8.8
223467_at	RASD1	8.8
1566472_s_at	RETSAT	8.7
201348_at	GPX3	8.6
1558420_at	C14orf180	8.6
209309_at	AZGP1	8.5
218062_x_at	CDC42EP4	8.3
204753_s_at	HLF	8.2
210096_at	CYP4B1	8.1
235129_at	PPP1R1A	8.1
210547_x_at	ICA1	8.0
218865_at	MOSC1	7.9
204755_x_at	HLF	7.7
225424_at	GPAM	7.7
242181_at	---	7.6
210130_s_at	TM7SF2	7.6
214721_x_at	CDC42EP4	7.6
203641_s_at	COBLL1	7.5
229309_at	ADRB1	7.5
212657_s_at	IL1RN	7.5
32625_at	NPR1	7.5
213486_at	COPG2IT1	7.4
203908_at	SLC4A4	7.3
205392_s_at	CCL14 /// CCL1	7.3
224461_s_at	AIFM2	7.2
1552615_at	ACACB	7.1
1553243_at	ITIH5	7.1
219298_at	ECHDC3	7.1
227190_at	TMEM37	7.0
220868_s_at	SLC7A10	6.9
204389_at	MAOA	6.9
207761_s_at	METTL7A	6.8
213855_s_at	LIPE	6.8
232313_at	TMEM132C	6.7
209581_at	PLA2G16	6.7
218810_at	ZC3H12A	6.6
239523_at	TUSC5	6.6
217626_at	AKR1C1	6.6
220287_at	ADAMTS9	6.5
206697_s_at	HP	6.5
1556989_at	---	6.4
1554485_s_at	TMEM37	6.4
226018_at	C7orf41	6.4
218665_at	FZD4	6.4
227856_at	C4orf32	6.3
206210_s_at	CETP	6.3
1559975_at	BTG1	6.3
200832_s_at	SCD	6.2
218736_s_at	PALMD	6.2
220437_at	LOC55908	6.2
222725_s_at	PALMD	6.2
211652_s_at	LBP	6.2
236923_x_at	---	6.1

204422_s_at	FGF2	6.0
218124_at	RETSAT	6.0
220736_at	SLC19A3	6.0
205295_at	CKMT2	5.8
214582_at	PDE3B	5.8
226448_at	FAM89A	5.8
238062_at	GPIHBP1	5.8
235382_at	AQPEP	5.8
201425_at	ALDH2	5.7
236656_s_at	LOC100288911	5.7
1562102_at	AKR1C1	5.7
206115_at	EGR3	5.6
228155_at	C10orf58	5.6
239345_at	SLC19A3	5.6
223427_s_at	EPB41L4B	5.6
204754_at	HLF	5.6
207275_s_at	ACSL1	5.5
232187_at	PALMD	5.5
1557832_at	---	5.5
209543_s_at	CD34	5.5
232662_x_at	C10orf58	5.5
230003_at	---	5.5
222139_at	KIAA1466	5.4
206737_at	WNT11	5.4
214038_at	CCL8	5.4
238718_at	---	5.4
32837_at	AGPAT2	5.4
241925_x_at	---	5.3
209369_at	ANXA3	5.3
224435_at	C10orf58	5.3
232882_at	---	5.2
823_at	CX3CL1	5.2
218966_at	MYO5C	5.2
218756_s_at	DHRS11	5.1
238003_at	HEPACAM	5.1
214434_at	HSPA12A	5.1
224480_s_at	AGPAT9	5.1
203722_at	ALDH4A1	5.1
228469_at	PPID	5.1
241368_at	PLIN5	5.1
236898_at	LOC100288781	5.0
212705_x_at	LOC100293124	5.0
201468_s_at	NQO1	5.0
1557116_at	APOL6	5.0
227417_at	MOSC2	4.9
206170_at	ADRB2	4.9
208707_at	EIF5	4.9
201236_s_at	BTG2	4.9
239001_at	MGST1	4.9
205208_at	ALDH1L1	4.9
203642_s_at	COBLL1	4.8
203608_at	ALDH5A1	4.8
206208_at	CA4	4.8

209074_s_at	FAM107A	4.8
