# Supplementary material for: Gene expression identifies heterogeneity of metastatic behavior among high-grade non-translocation associated soft tissue sarcomas
Source: J Transl Med. 2014 Jun 20;12:176. doi: 10.1186/1479-5876-12-176 (PMC4082412; doi:10.1186/1479-5876-12-176)
Supplement: Additional file 2 — Genes over-expressed in LipoD-A vs LipoD-B. [file 1479-5876-12-176-S2.zip › Table 2B.pdf]

Table 2B	Genes over-expressed in LipoD-B vs LipoD-A	
Probe id	Gene Symbol	Fold Change (Up in LipoD-B vs LipoD-A)
232231_at	RUNX2	26.4
229802_at	---	23.1
203184_at	FBN2	19.5
209773_s_at	RRM2	14.8
204035_at	SCG2	14.3
202870_s_at	CDC20	14.2
212473_s_at	MICAL2	13.2
201291_s_at	TOP2A	12.3
212472_at	MICAL2	12.0
1554474_a_at	MOXD1	11.6
235821_at	---	11.3
213358_at	KIAA0802	11.3
1552619_a_at	ANLN	10.2
1555758_a_at	CDKN3	10.1
222608_s_at	ANLN	10.1
235629_at	---	9.9
214702_at	FN1	9.7
205347_s_at	TMSB15A	9.7
219918_s_at	ASPM	9.4
230424_at	C5orf13	9.3
225655_at	UHRF1	9.2
225387_at	TSPAN5	9.0
209408_at	KIF2C	8.9
215077_at	---	8.8
221558_s_at	LEF1	8.6
220955_x_at	RAB23	8.6
202954_at	UBE2C	8.5
218755_at	KIF20A	8.1
214701_s_at	FN1	8.0
225681_at	CTHRC1	7.9
203214_x_at	CDK1	7.9
202503_s_at	KIAA0101	7.8
209890_at	TSPAN5	7.8
206364_at	KIF14	7.7
210052_s_at	TPX2	7.7
225687_at	FAM83D	7.6
201292_at	TOP2A	7.5
208079_s_at	AURKA	7.3
214710_s_at	CCNB1	7.3
204825_at	MELK	7.3
209714_s_at	CDKN3	7.3
204298_s_at	LOX	7.3
223805_at	OSBPL6	7.1
201890_at	RRM2	7.1
232278_s_at	DEPDC1	7.0
210559_s_at	CDK1	6.9
209642_at	BUB1	6.8
223381_at	NUF2	6.8
229256_at	PGM2L1	6.7
223229_at	UBE2T	6.6

204822_at	TTK	6.6
228729_at	CCNB1	6.5
228273_at	---	6.5
1558199_at	FN1	6.5
203764_at	DLGAP5	6.5
202705_at	CCNB2	6.4
203755_at	BUB1B	6.3
207828_s_at	CENPF	6.3
204170_s_at	CKS2	6.2
213226_at	CCNA2	6.0
202728_s_at	LTBP1	6.0
205122_at	TMEFF1	6.0
204962_s_at	CENPA	6.0
204444_at	KIF11	5.9
210135_s_at	SHOX2	5.9
228293_at	DEPDC7	5.8
222344_at	---	5.7
242052_at	---	5.7
235545_at	DEPDC1	5.6
218820_at	C14orf132	5.6
227801_at	TRIM59	5.6
219306_at	KIF15	5.6
219493_at	SHCBP1	5.4
222958_s_at	DEPDC1	5.4
1554768_a_at	MAD2L1	5.4
228323_at	CASC5	5.3
204092_s_at	AURKA	5.3
204709_s_at	KIF23	5.3
218662_s_at	NCAPG	5.3
203554_x_at	PTTG1	5.2
222039_at	KIF18B	5.2
214595_at	KCNG1	5.2
226658_at	PDPN	5.1
218663_at	NCAPG	5.1
1554466_a_at	C16orf13	5.0
218051_s_at	NT5DC2	5.0
235476_at	TRIM59	5.0
229490_s_at	---	4.9
230165_at	SGOL2	4.9
218349_s_at	ZWILCH	4.9
214806_at	BICD1	4.9
203418_at	CCNA2	4.8
1559394_a_at	---	4.8
231964_at	---	4.7
202733_at	P4HA2	4.7
1556051_a_at	BICD1	4.7
233036_at	---	4.6
218726_at	HJURP	4.6
213435_at	SATB2	4.5
1556543_at	---	4.5
219249_s_at	FKBP10	4.5
225735_at	ANKRD50	4.5
232094_at	C15orf29	4.4

208443_x_at	SHOX2	4.4
206102_at	GINS1	4.4
223392_s_at	TSHZ3	4.4
203196_at	ABCC4	4.3
207265_s_at	KDELR3	4.3
221011_s_at	LBH	4.2
213007_at	FANCI	4.2
218979_at	RMI1	4.2
209035_at	MDK	4.2
219787_s_at	ECT2	4.2
205053_at	PRIM1	4.2
203362_s_at	MAD2L1	4.2
209765_at	ADAM19	4.2
229442_at	C18orf54	4.2
1560813_at	---	4.1
223463_at	RAB23	3.9
228843_at	---	3.9
229097_at	DIAPH3	3.9
212481_s_at	TPM4	3.9
222610_s_at	S100PBP	3.8
229553_at	PGM2L1	3.8
201430_s_at	DPYSL3	3.8
1557455_s_at	MOSPD1	3.8
209464_at	AURKB	3.8
238478_at	BNC2	3.8
204240_s_at	SMC2	3.8
232568_at	MGC24103	3.8
219410_at	TMEM45A	3.7
223274_at	TCF19	3.7
225898_at	WDR54	3.7
229784_at	MGC16121	3.7
226826_at	---	3.7
207124_s_at	GNB5	3.7
201309_x_at	C5orf13	3.7
235723_at	BNC2	3.7
230722_at	BNC2	3.7
214845_s_at	CALU	3.7
205034_at	CCNE2	3.6
208119_s_at	ZNF93	3.6
1567107_s_at	TPM4	3.6
203370_s_at	PDLIM7	3.5
204017_at	KDELR3	3.5
222587_s_at	GALNT7	3.5
212464_s_at	FN1	3.5
227249_at	NDE1	3.5
203022_at	RNASEH2A	3.4
239091_at	---	3.4
201774_s_at	NCAPD2	3.4
207357_s_at	GALNT10	3.4
201663_s_at	SMC4	3.4
226899_at	UNC5B	3.4
210220_at	FZD2	3.4
231859_at	C14orf132	3.4

202613_at	CTPS	3.3
226421_at	AMMECR1	3.3
219376_at	ZNF322B	3.3
229610_at	CKAP2L	3.3
232816_s_at	DDX11	3.3
220272_at	BNC2	3.3
238549_at	CBFA2T2	3.3
215489_x_at	HOMER3	3.2
216952_s_at	LMNB2	3.2
229422_at	NRD1	3.2
206613_s_at	TAF1A	3.2
221922_at	GPSM2	3.2
222077_s_at	RACGAP1	3.2
209344_at	TPM4	3.2
209927_s_at	C1orf77	3.2
210495_x_at	FN1	3.2
216442_x_at	FN1	3.1
225961_at	KLHDC5	3.1
229333_at	---	3.1
243816_at	ZNF70	3.1
229942_at	BNC2	3.1
227628_at	GPX8	3.1
223393_s_at	TSHZ3	3.0
218741_at	CENPM	3.0
200756_x_at	CALU	3.0
217771_at	GOLM1	3.0
224002_s_at	FKBP7	3.0
218313_s_at	GALNT7	3.0
235925_at	---	3.0
211719_x_at	FN1	3.0
204976_s_at	AMMECR1	3.0
228620_at	---	3.0
232344_at	---	3.0
210983_s_at	MCM7	2.9
217755_at	HN1	2.9
212624_s_at	CHN1	2.9
215629_s_at	DLEU2 /// DLE	2.9
205339_at	STIL	2.9
227220_at	NFXL1	2.9
1555793_a_at	ZFP82	2.9
242228_at	---	2.9
230229_at	DLG1	2.9
201564_s_at	FSCN1	2.9
203390_s_at	KIF3C	2.9
