# Supplementary material for: Gene expression identifies heterogeneity of metastatic behavior among high-grade non-translocation associated soft tissue sarcomas
Source: J Transl Med. 2014 Jun 20;12:176. doi: 10.1186/1479-5876-12-176 (PMC4082412; doi:10.1186/1479-5876-12-176)
Supplement: Additional file 3 — Genes over-expressed in UPS-A vs UPS-B. [file 1479-5876-12-176-S3.zip › Table 3A.pdf]

Table 3A	Genes over-expressed in UPS-A vs UPS-B	
Probe id	Gene Symbol	Fold Change (Up in UPS-A vs UPS-B)
235849_at	SCARA5	34.0
229839_at	SCARA5	33.8
204719_at	ABCA8	29.1
206002_at	GPR64	18.2
232267_at	GPR133	17.0
227899_at	VIT	16.3
213451_x_at	TNXA /// TNXB	14.8
205200_at	CLEC3B	14.7
216333_x_at	TNXA /// TNXB	14.7
206093_x_at	TNXA /// TNXB	13.5
208609_s_at	TNXB	12.7
223075_s_at	AIF1L	11.8
228854_at	---	11.5
236184_at	---	11.3
203180_at	ALDH1A3	11.2
242541_at	ABCA9	10.6
209612_s_at	ADH1B	10.6
219230_at	TMEM100	9.9
209613_s_at	ADH1B	9.0
243357_at	NEGR1	9.0
229461_x_at	NEGR1	8.4
213764_s_at	MFAP5	8.0
213765_at	MFAP5	7.9
209343_at	EFHD1	7.9
230418_s_at	GALNTL1	7.5
219304_s_at	PDGFD	7.1
227762_at	---	7.1
244885_at	---	7.1
228368_at	ARHGAP20	7.0
212327_at	LIMCH1	6.9
216339_s_at	TNXA /// TNXB	6.9
212328_at	LIMCH1	6.9
205883_at	ZBTB16	6.4
229357_at	ADAMTS5	6.4
205150_s_at	TRIL	6.2
219295_s_at	PCOLCE2	6.1
207414_s_at	PCSK6	6.1
239370_at	---	6.1
201525_at	APOD	6.0
212325_at	LIMCH1	6.0
227530_at	AKAP12	5.9
205151_s_at	TRIL	5.9
227529_s_at	AKAP12	5.8
210397_at	DEFB1	5.8
204731_at	TGFBR3	5.8
217504_at	ABCA6	5.7
240188_at	---	5.5
219935_at	ADAMTS5	5.5
226625_at	TGFBR3	5.5
219059_s_at	LYVE1	5.4

202191_s_at	GAS7	5.4
221795_at	NTRK2	5.3
238066_at	RBP7	5.3
212713_at	MFAP4	5.3
205083_at	AOX1	5.2
200965_s_at	ABLIM1	5.2
1558636_s_at	ADAMTS5	5.2
227662_at	SYNPO2	5.2
227997_at	IL17RD	5.1
220392_at	EBF2	5.1
229266_at	SHISA6	5.1
227419_x_at	PLAC9	5.0
235079_at	---	5.0
232555_at	CREB5	5.0
225207_at	PDK4	5.0
227148_at	PLEKHH2	5.0
235368_at	ADAMTS5	4.9
217525_at	OLFML1	4.9
220751_s_at	C5orf4	4.9
203859_s_at	PALM	4.8
202192_s_at	GAS7	4.8
205931_s_at	CREB5	4.8
205794_s_at	NOVA1	4.8
228434_at	BTNL9	4.8
229487_at	EBF1	4.8
220266_s_at	KLF4	4.8
1556427_s_at	LRRN4CL	4.7
213568_at	OSR2	4.7
229228_at	CREB5	4.7
239185_at	ABCA9	4.7
231382_at	FGF18	4.6
220037_s_at	LYVE1	4.6
231067_s_at	AKAP12	4.6
205382_s_at	CFD	4.6
213661_at	PAMR1	4.5
244876_at	---	4.5
216910_at	XPNPEP2	4.5
32625_at	NPR1	4.5
219871_at	FLJ13197	4.5
242342_at	---	4.5
229310_at	KLHL29	4.5
223366_at	---	4.4
210872_x_at	GAS7	4.4
209789_at	CORO2B	4.4
233261_at	EBF1	4.4
243584_at	---	4.4
225525_at	KIAA1671	4.3
214433_s_at	SELENBP1	4.3
227550_at	GFRA1	4.3
238091_at	---	4.3
206030_at	ASPA	4.3
205082_s_at	AOX1	4.2
226096_at	FNDC5	4.2

242662_at	PCSK6	4.2
225895_at	SYNPO2	4.2
207704_s_at	GAS7	4.2
204396_s_at	GRK5	4.2
203908_at	SLC4A4	4.2
214890_s_at	FAM149A	4.1
240245_at	---	4.1
201348_at	GPX3	4.1
235570_at	RBMS3	4.1
210096_at	CYP4B1	4.1
219778_at	ZFPM2	4.1
241459_at	---	4.1
226250_at	---	4.0
214091_s_at	GPX3	4.0
211067_s_at	GAS7	4.0
205364_at	ACOX2	4.0
223749_at	C1QTNF2	4.0
227058_at	C13orf33	4.0
209821_at	IL33	4.0
236335_at	---	4.0
230744_at	FSTL1	4.0
227646_at	EBF1	4.0
239262_at	---	3.9
225627_s_at	CACHD1	3.9
226901_at	C17orf58	3.9
206484_s_at	XPNPEP2	3.9
229985_at	---	3.8
1553194_at	NEGR1	3.8
210517_s_at	AKAP12	3.8
232204_at	EBF1	3.8
227875_at	KLHL13	3.8
226627_at	8-Sep	3.7
231024_at	LOC572558	3.7
221016_s_at	TCF7L1	3.7
204395_s_at	GRK5	3.7
213005_s_at	KANK1	3.7
222830_at	GRHL1	3.7
227082_at	---	3.7
203851_at	IGFBP6	3.7
1562477_at	EBF2	3.7
210471_s_at	KCNAB1	3.7
227717_at	ARHGEF37	3.7
218901_at	PLSCR4	3.6
228580_at	HTRA3	3.6
230417_at	GALNTL1	3.6
213075_at	OLFML2A	3.6
233520_s_at	CMYA5	3.6
209098_s_at	JAG1	3.6
238512_at	---	3.6
226950_at	ACVRL1	3.6
229004_at	ADAMTS15	3.5
219686_at	STK32B	3.5
242605_at	---	3.5

223315_at	NTN4	3.5
212486_s_at	FYN	3.5
240865_at	---	3.5
216840_s_at	LAMA2	3.5
226188_at	HSPC159	3.5
227410_at	FAM43A	3.5
212558_at	SPRY1	3.5
235174_s_at	LOC100128822	3.4
243041_s_at	---	3.4
210078_s_at	KCNAB1	3.4
210702_s_at	PTGIS	3.4
235538_at	---	3.4
49452_at	ACACB	3.4
205501_at	PDE10A	3.4
235182_at	ISM1	3.4
206170_at	ADRB2	3.4
226985_at	FGD5	3.4
1560049_at	---	3.3
205384_at	FXYD1	3.3
211892_s_at	PTGIS	3.3
1561690_at	---	3.3
222860_s_at	PDGFD	3.3
214761_at	ZNF423	3.3
229084_at	CNTN4	3.3
225016_at	APCDD1	3.3
227526_at	CDON	3.3
209543_s_at	CD34	3.3
240815_at	---	3.3
41644_at	SASH1	3.3
224976_at	NFIA	3.2
234104_at	---	3.2
1558579_at	FLJ37786	3.2
235318_at	FBN1	3.2
203788_s_at	SEMA3C	3.2
206987_x_at	FGF18	3.2
227121_at	---	3.2
232406_at	---	3.2
230958_s_at	---	3.2
214295_at	KIAA0485	3.2
238029_s_at	SLC16A14	3.2
226806_s_at	NFIA	3.2
229222_at	ACSS3	3.2
231183_s_at	JAG1	3.1
227243_s_at	EBF3	3.1
