# Supplementary material for: Gene expression identifies heterogeneity of metastatic behavior among high-grade non-translocation associated soft tissue sarcomas
Source: J Transl Med. 2014 Jun 20;12:176. doi: 10.1186/1479-5876-12-176 (PMC4082412; doi:10.1186/1479-5876-12-176)
Supplement: Additional file 3 — Genes over-expressed in UPS-A vs UPS-B. [file 1479-5876-12-176-S3.zip › Table 3B.pdf]

Table 3B	Genes over-expressed in UPS-B vs UPS-A	
Probe id	Gene Symbol	Fold Change (Up in UPS-B vs UPS-A)
205542_at	STEAP1	19.4
37892_at	COL11A1	18.9
213909_at	LRRC15	18.5
213338_at	TMEM158	17.2
212942_s_at	KIAA1199	14.8
203963_at	CA12	14.8
206513_at	AIM2	14.3
201645_at	TNC	12.1
219985_at	HS3ST3A1	11.1
218469_at	GREM1	10.9
212472_at	MICAL2	10.8
215867_x_at	CA12	10.8
204320_at	COL11A1	10.7
203936_s_at	MMP9	10.6
214164_x_at	CA12	10.2
203434_s_at	MME	9.9
229479_at	---	9.9
209924_at	CCL18	9.5
205959_at	MMP13	9.5
32128_at	CCL18	9.2
212473_s_at	MICAL2	8.7
218468_s_at	GREM1	8.5
210735_s_at	CA12	8.2
204508_s_at	CA12	8.2
204475_at	MMP1	8.1
227140_at	---	8.0
232231_at	RUNX2	7.8
210511_s_at	INHBA	7.4
223278_at	GJB2	7.1
238617_at	---	7.1
203889_at	SCG5	7.1
223484_at	C15orf48	6.9
204337_at	RGS4	6.4
201850_at	CAPG	6.4
222877_at	---	6.3
205798_at	IL7R	6.2
205941_s_at	COL10A1	6.2
216005_at	TNC	6.2
228481_at	---	6.2
226218_at	IL7R	6.0
226777_at	---	5.9
1555778_a_at	POSTN	5.8
236859_at	RUNX2	5.6
205681_at	BCL2A1	5.5
228658_at	MIAT	5.5
202952_s_at	ADAM12	5.5
225681_at	CTHRC1	5.5
219386_s_at	SLAMF8	5.5
231867_at	ODZ2	5.5
206134_at	ADAMDEC1	5.4

217428_s_at	COL10A1	5.4
219257_s_at	SPHK1	5.3
213790_at	ADAM12	5.3
208637_x_at	ACTN1	5.2
204254_s_at	VDR	5.2
204638_at	ACP5	5.2
206766_at	ITGA10	5.2
203435_s_at	MME	5.2
214866_at	PLAUR	5.1
218051_s_at	NT5DC2	5.1
204006_s_at	FCGR3A /// FC	5.1
218788_s_at	SMYD3	4.9
229225_at	NRP2	4.9
204268_at	S100A2	4.9
208636_at	ACTN1	4.8
233690_at	---	4.8
219725_at	TREM2	4.8
235821_at	---	4.8
205242_at	CXCL13	4.7
225387_at	TSPAN5	4.7
204675_at	SRD5A1	4.6
221491_x_at	HLA-DRB1 /// H	4.6
227828_s_at	FAM176A	4.6
221558_s_at	LEF1	4.6
229437_at	MIR155HG	4.6
216834_at	RGS1	4.5
206932_at	CH25H	4.5
236858_s_at	---	4.5
202274_at	ACTG2	4.5
204285_s_at	PMAIP1	4.5
219179_at	DACT1	4.4
202833_s_at	SERPINA1	4.3
219385_at	SLAMF8	4.3
202988_s_at	RGS1	4.3
227361_at	HS3ST3B1	4.3
234994_at	TMEM200A	4.3
209514_s_at	RAB27A	4.2
210951_x_at	RAB27A	4.2
209604_s_at	GATA3	4.1
223158_s_at	NEK6	4.1
209890_at	TSPAN5	4.1
217996_at	PHLDA1	4.0
228821_at	ST6GAL2	4.0
239893_at	---	4.0
219874_at	SLC12A8	4.0
236044_at	PPAPDC1A	4.0
210809_s_at	POSTN	4.0
220918_at	C21orf96	4.0
211160_x_at	ACTN1	4.0
209823_x_at	HLA-DQB1	4.0
229635_at	---	4.0
224823_at	MYLK	3.9
214981_at	POSTN	3.9

212998_x_at	HLA-DQB1 /// L	3.9
202934_at	HK2	3.9
220658_s_at	ARNTL2	3.8
203083_at	THBS2	3.8
207030_s_at	CSRP2	3.8
210889_s_at	FCGR2B	3.8
209606_at	CYTIP	3.8
209765_at	ADAM19	3.8
202555_s_at	MYLK	3.7
204286_s_at	PMAIP1	3.7
226372_at	CHST11	3.7
209301_at	CA2	3.7
209875_s_at	SPP1	3.7
214632_at	NRP2	3.7
210845_s_at	PLAUR	3.6
202206_at	ARL4C	3.6
211924_s_at	PLAUR	3.6
218424_s_at	STEAP3	3.6
209955_s_at	FAP	3.6
214702_at	FN1	3.6
212543_at	AIM1	3.6
205214_at	STK17B	3.6
225258_at	FBLIM1	3.5
204255_s_at	VDR	3.5
232701_at	---	3.5
203823_at	RGS3	3.5
226223_at	---	3.5
204446_s_at	ALOX5	3.4
241026_at	---	3.4
238439_at	ANKRD22	3.4
235122_at	HIVEP3	3.4
211656_x_at	HLA-DQB1 /// L	3.4
1555480_a_at	FBLIM1	3.4
212657_s_at	IL1RN	3.3
204004_at	PAWR	3.3
209773_s_at	RRM2	3.3
201422_at	IFI30	3.3
207173_x_at	CDH11	3.3
204774_at	EVI2A	3.3
215199_at	CALD1	3.3
225566_at	NRP2	3.3
209238_at	STX3	3.3
221530_s_at	BHLHE41	3.3
209218_at	SQLE	3.3
207536_s_at	TNFRSF9	3.3
226997_at	ADAMTS12	3.2
224204_x_at	ARNTL2	3.2
201286_at	SDC1	3.2
209803_s_at	PHLDA2	3.2
207172_s_at	CDH11	3.2
207265_s_at	KDELR3	3.1
204079_at	TPST2	3.1
211654_x_at	HLA-DQB1	3.1

218499_at	MST4	3.1
227801_at	TRIM59	3.1
203196_at	ABCC4	3.1
217997_at	PHLDA1	3.1
214511_x_at	FCGR1B	3.1
212624_s_at	CHN1	3.1
204005_s_at	PAWR	3.1
201287_s_at	SDC1	3.1
225436_at	FAM108C1	3.1
219557_s_at	NRIP3	3.1
211395_x_at	FCGR2C	3.1
201313_at	ENO2	3.0
203710_at	ITPR1	3.0
211429_s_at	SERPINA1	3.0
228293_at	DEPDC7	3.0
243366_s_at	---	3.0
216236_s_at	SLC2A14 /// SL	3.0
1555756_a_at	CLEC7A	3.0
226099_at	ELL2	3.0
203764_at	DLGAP5	3.0
220147_s_at	FAM60A	3.0
202497_x_at	SLC2A3	3.0
213577_at	SQLE	3.0
229055_at	GPR68	3.0
223767_at	GPR84	3.0
202619_s_at	PLOD2	2.9
218368_s_at	TNFRSF12A	2.9
228964_at	PRDM1	2.9
242465_at	---	2.9
225646_at	CTSC	2.9
235476_at	TRIM59	2.9
206060_s_at	PTPN22	2.9
201695_s_at	PNP	2.9
223172_s_at	MTP18	2.9
223159_s_at	NEK6	2.9
222088_s_at	SLC2A14 /// SL	2.9
219634_at	CHST11	2.9
230391_at	CD84	2.9
217127_at	CTH	2.9
1552485_at	LACTB	2.9
202620_s_at	PLOD2	2.9
218211_s_at	MLPH	2.9
216950_s_at	FCGR1A /// FC	2.9
210992_x_at	FCGR2C	2.8
