# Supplementary material for: Gene expression identifies heterogeneity of metastatic behavior among high-grade non-translocation associated soft tissue sarcomas
Source: J Transl Med. 2014 Jun 20;12:176. doi: 10.1186/1479-5876-12-176 (PMC4082412; doi:10.1186/1479-5876-12-176)
Supplement: Additional file 4 — Genes over-expressed in both LipoD-B vs LipoD-A and UPS-B vs UPS-A. [file 1479-5876-12-176-S4.pdf]

Genes over-expressed in both LipoD-B vs LipoD-A and UPS-B vs UPS-A

Gene Symbol	Fold Change (Up in LipoD-B vs LipoD-A)	Fold Change (Up in UPS-B vs UPS-A)
TSPAN5	9.0	4.7
TRIM59	5.6	3.1
SQLE	2.7	3.3
RUNX2	26.4	7.8
RRM2	14.8	3.3
RAB27A	2.5	4.2
MICAL2	13.2	10.9
LEF1	8.6	4.6
KDELR3	4.3	3.1
FZD2	3.4	2.6
FN1	9.8	3.6
CTHRC1	7.9	5.5
CHN1	2.9	3.1
ADAM19	4.2	3.8
ABCC4	4.3	3.1
